# Known but unpredictable – an argument for complexity

**DOI:** 10.1192/bjb.2018.12

**Published:** 2018-04

**Authors:** Martin Plöderl, Clemens Fartacek

**Affiliations:** Department of Clinical Psychology and Department of Crisis Intervention and Suicide Prevention, Paracelsus Medical University, Salzburg, Austria; email: m.ploederl@salk.at

Since the seminal paper of Pokorny in 1983,^1^ the prediction of suicides has not improved, as Large *et al* have pointed out in their current paper^2^ and in previous meta-analyses.^3^^–^^7^ In opposition to most current recommendations in suicide prevention, which still require clinicians to formulate levels of suicide risk,^8^ Large *et al*^2^ suggest that clinicians should give up risk formulation and instead focus directly on the individual needs of patients to deliver optimal care. They argue that uncertainty in the prediction of suicide is largely aleatory (dependent on random processes) and also epistemic (lacking knowledge). We think that one important explanation is missing: complexity.

Complexity refers to behaviours produced by nonlinear dynamic systems, which cannot be predicted in the long term, even if the generating system operates completely deterministically and is known in detail. The most prominent type of complex dynamics is deterministic chaos, which became familiar as the ‘butterfly effect’. During chaotic dynamics, even the smallest differences in initial conditions lead to a massive divergence of trajectories over time. Owing to complex behaviours such as chaos, from a nonlinear dynamical perspective, the failure of long-term predictions of suicidal behaviour could be a consequence not only of incomplete epistemic knowledge (e.g. unspecific or unknown risk factors) or aleatory processes (random noise), but also of the inherent complexity of the underlying system.

Are there any alternatives for predicting suicidal behaviour from a nonlinear dynamical perspective? Natural sciences (e.g. geophysics) have developed methods for the short-term prediction of extreme events (e.g. tsunamis), based on continuous monitoring of appropriate signals and identification of nonlinear dynamical precursors.^9^^,^^10^ This might be a promising approach for suicide research as well. Given the recent improvements of scientific methods, an empirical application of complexity theory in suicide research seems realistic.^11^^,^^12^ However, it still has to be demonstrated that such novel approaches are feasible in clinical practice and that they can in fact improve the prediction of suicides.

We believe that suicidology needs to take complexity theory into consideration. If not, much time, effort and money will continue to go into approaches that, from the viewpoint of complexity theory, lead to a dead end. This includes the search for novel risk factors or combinations of risk factors (e.g. by applying machine learning) without acknowledging the underlying complex processes.
